# Serum arterial lactate at the time of admission as a predictor of mortality in patients admitted with severe sepsis and septic shock to an ICU

**DOI:** 10.1186/cc12947

**Published:** 2013-11-05

**Authors:** Adriell Ramalho Santana, Fábio Ferreira Amorim, Bárbara Magalhães Menezes, Felipe Bozi Soares, Fernanda Vilas Bôas Araújo, Jacqueline Lima de Souza, Mariana Pinheiro Barbosa de Araújo, Louise Cristhine de Carvalho Santos, Pedro Henrique Gomes Rocha, Osvaldo Gonçalves da Silva Neto, Guilherme Menezes de Andrade Filho, Pedro Nery Ferreira Júnior, Alethea Patrícia Pontes Amorim, Rodrigo Santos Biondi, Rubens Antônio Bento Ribeiro

**Affiliations:** 1 Escola Superior de Ciências da Saúde, Brasília, Brazil; 2 Liga Acadêmica de Medicina Intensiva de Brasília, Brazil; 3 Hospital Anchieta, Brasília, Brazil

## Background

Elevated serum arterial lactate levels are often associated with an imbalance between oxygen demand and delivery, which has a strong correlation with poorer outcomes in critically ill patients [1,2]. This study aims to evaluate serum arterial lactate as a predictor of mortality in critical patients admitted with severe sepsis and septic shock.

## Materials and methods

Retrospective cohort study conducted in the ICU of Hospital Anchieta, Brasília, DF, Brazil, during 3 years. For the first analysis, patients were divided into two groups: group with arterial lactate >2 mmol/l and group with low arterial lactate ≤2 mmol/l at the time of admission. For a second analysis, patients were divided into two groups: group with arterial lactate >3.3 mmol/l and group with arterial lactate ≤3.3 mmol/l at the time of admission.

## Results

A total of 195 patients with sepsis were enrolled, 41% (*n *= 80) with septic shock. Mean age was 63 ± 22 years, ICU length of stay 9 ± 11 days, SAPS3 62 ± 16, and APACHE II 21 ± 9. ICU mortality in 4 days was 10.8% (*n *= 21), in 28 days was 12.3% (*n *= 24), and hospital mortality was 26.2% (*n *= 51). The nonsurvivor patients had higher lactate values (2.0 ± 1.4 vs. 1.3 ± 1.1, *P *= 0.00). Considering the arterial lactate cutoff value of 2.0 mmol/l, there was no difference between groups regarding ICU length of stay (10 ± 13 vs. 9 ± 2 days, *P *= 0.47), mortality in 4 days (12% vs. 10%, *P *= 0.85), mortality in 28 days (13% vs. 16%, *P *= 0.77), and hospital mortality (30% vs. 32%, *P *= 0.86). However, considering the lactate cutoff value of 3.3 mmol/l, the high lactate group had higher mortality in 4 days (27% vs. 9%, *P *= 0.04) and hospital mortality (67% vs. 23%, *P *= 0.00). There was no statistical significant difference regarding mortality in 28 days (27% vs. 11%, *P *= 0.08), and ICU length of stay (8 ± 7 vs. 9 ± 11 days, *P *= 0.59). The relative risk of hospital death in patients with arterial lactate >3.3 mmol/l was 2.93 (95% CI: 1.87 to 4.58). The specificity of arterial lactate >3.3 mmol/l for hospital mortality was 96.5% (95% CI: 92.1 to 98.5%), sensibility was 19.6% (95% CI: 11.0 to 32.5%), and LR+ was 5.65 (95% CI: 2.03 to 15.7%). The arterial lactate area under the ROC curve for mortality was 0.634 (95% CI: 0.540 to 0.748).

## Conclusions

In the patients admitted with severe sepsis and septic shock for this sample, the nonsurvivors had higher lactate values. Arterial lactate >2 mmol/l at the time of admission was not associated with mortality. Arterial lactate >3.3 mmol/l was associated with mortality in 4 days, and hospital mortality. Indeed, lactate >3.3 mmol/l had high specificity for hospital mortality.
